# Optical Passive Sensor Calibration for Satellite Remote Sensing and the Legacy of NOAA and NIST Cooperation

**DOI:** 10.6028/jres.119.008

**Published:** 2014-06-24

**Authors:** Raju Datla, Michael Weinreb, Joseph Rice, B. Carol Johnson, Eric Shirley, Changyong Cao

**Affiliations:** 1 NOAA Affiliate, ERT, Inc., Laurel, MD 20707; 2 NOAA NSOF/E/OSD, Silver Spring, MD 20910; 3 National Institute of Standards and Technology, Gaithersburg, MD 20899; 4 NOAA/NESDIS/STAR, College Park, MD 20737

**Keywords:** climate change, integrating sphere, irradiance standard, ocean color, radiance standard, radiometry, remote sensing sensor calibration, weather satellites

## Abstract

This paper traces the cooperative efforts of scientists at the National Oceanic and Atmospheric Administration (NOAA) and the National Institute of Standards and Technology (NIST) to improve the calibration of operational satellite sensors for remote sensing of the Earth’s land, atmosphere and oceans. It gives a chronological perspective of the NOAA satellite program and the interactions between the two agencies’ scientists to address pre-launch calibration and issues of sensor performance on orbit. The drive to improve accuracy of measurements has had a new impetus in recent years because of the need for improved weather prediction and climate monitoring. The highlights of this cooperation and strategies to achieve SI-traceability and improve accuracy for optical satellite sensor data are summarized^1^.

## 1. Introduction

From the beginning of the era of meteorological satellites, a remarkable goal-oriented interaction between scientists and engineers of the National Oceanic and Atmospheric Administration (NOAA), the National Aeronautics and Space Administration (NASA) and the National Institute of Standards and Technology (NIST) (formerly the National Bureau of Standards [NBS]) has been taking place to reduce measurement uncertainties and improve the quality of observations. Reports and papers published in the literature document this effort. This paper traces the NOAA and NIST cooperation for eliminating the discrepancies in the measurements of NOAA operational sensors in orbit and developing strategies to enable their radiometric calibration to meet international standards, with the ultimate goal of improved weather prediction and climate change monitoring. It gives a chronological perspective of the growth of the cooperation between the agencies, rather than a specific technical discussion. The section, “Legacy of the Early Interval,” discusses the events at the beginnings of the weather satellite program and the development of the state of the art with respect to calibration methodology. Both NASA and NOAA worked with National Bureau of Standards (now NIST) for the development of advanced lamp standards, radiometers, artifact standards and methodologies for calibrations of space-bound optical sensors and their components to quantify and reduce uncertainties in remote sensing data. These capabilities enabled other satellite launching agencies, such as the Department of Defense (DOD) and the United States Geological Society (USGS), to support and use NIST for the characterization and pre-launch calibration of sensors to be launched into space. The following section, “Legacy of Recent Interactions,” covers the cooperation between NOAA and NIST to address issues connected to the calibration of NOAA’s operational sensors to meet the current goals at NOAA for improved weather prediction and climate monitoring. A similar collaborative effort between NASA and NIST, preceding this work, was established in the 90s to support NASA’s Earth Observing System (EOS). This collaboration provided the foundation of NIST’s modern capabilities and facilitated the NOAA/NIST work described here. NASA continued to provide a supporting role for the important achievements in the NOAA and NIST cooperation described in Sec. 3.5.

## 2. Legacy of the Early Interval

**Fig. 1:**
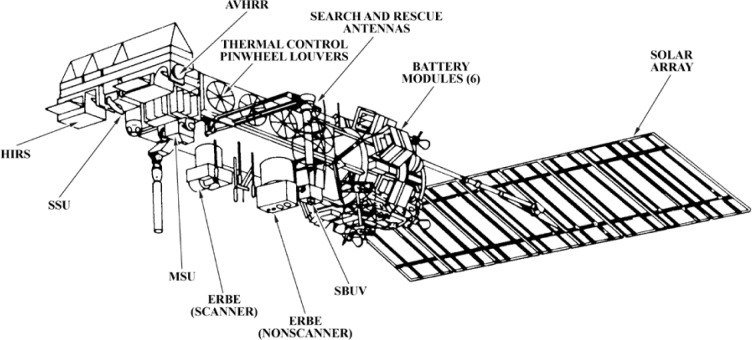
The nominal configuration of the POES ATN satellites (source: “Weather Satellites” American Meteorological Society, Boston 1990 [2]).

From the beginning NASA has been in the forefront of developing the technology for satellite-based remote sensing, and NOAA has worked with NASA to apply this technology for observing the weather and the global environment. The satellite series, Television Infrared Observation Satellite (TIROS), starting in 1960, and NASA Meteorological satellite (Nimbus), starting in 1964, were launched and operated over many years with the participation of scientists from both agencies. These programs advanced the technology used to observe the weather and the environment [1,2]. By 1970 the technology had matured enough for routine weather observations, and the Polar-orbiting Operational Environmental satellite (POES) series started with NOAA-1 as a second-generation weather satellite operated by NOAA’s National Environmental Satellite Service (NESS), now the National Environmental Satellite, Data, and Information Service (NESDIS), located in Suitland, Maryland. In 1978, the TIROS-N (N for the next-generation) series of POES was initiated, which consisted of a sequence of three satellites—TIROS-N, NOAA-6, and NOAA-7. In 1983 NOAA began the Advanced TIROS-N (ATN) series, consisting of NOAA-8 and subsequent satellites [2]. There were two primary sensors in the TIROS-N and ATN series. One was the Advanced Very High Resolution Radiometer (AVHRR), shown on the satellite in Fig. 1. The AVHRR had 4 channels in the visible and the thermal infrared portions of the spectrum and provided measurements of radiance. The data were applied to monitoring clouds, snow, and sea surface temperature, etc. The second was the TIROS Operational Vertical Sounder (TOVS) which consisted of three subsystems, the High Resolution Infrared Radiation Sounder (HIRS), the Stratospheric Sounding Unit (SSU) and the Microwave Sounding Unit (MSU), which on later satellites was replaced by the Advanced Microwave Sounder (AMSU). The TOVS suite was designed to make measurements in the infrared and microwave regions to provide data on temperature profiles from the surface to 1 kPa, water vapor content, and the total ozone content. In addition, NOAA-9 (launched in 1984) and NOAA-10 (launched in 1986) carried the Earth Radiation Budget Experiment (ERBE) instruments, designed to measure monthly and seasonal balance of radiation incident on and emitted by the Earth-atmosphere system, and the effect of clouds on the radiation budget. The original ERBE was carried on a NASA satellite [(ERBS—Earth Radiation Budget Satellite)] launched from the Space Shuttle in 1984. ERBE had two parts, the first, a nadir-viewer having five channels, four to view the earth and the fifth to view the sun. The second part of ERBE scanned across the earth from limb to limb with three channels, one to isolate the shortwave, the second to cover long wave and the third to cover the total radiation. Also, since 1984 the POES ATN satellites have carried a Solar Backscatter Ultraviolet instrument (SBUV/2) to measure backscatter in the 160 nm to 400 nm range to determine ozone profiles and total ozone.

### 2.1 The Coastal Zone Color Scanner (CZCS)

NESS became involved in ocean color measurements in the late 70s through Warren Hovis, formerly of NASA’s Goddard Space Flight Center (GSFC). Hovis, with colleagues at NASA, developed a satellite sensor, the Coastal Zone Color Scanner (CZCS), which was flown on Nimbus 7 in 1978. AVHRR and CZCS sensors proved capable of providing reliable data. Scientists then faced the challenge to radiometrically calibrate the sensors and improve their on-orbit radiometric accuracy.

The launch of CZCS in 1978 for ocean color measurements was based on many observations by NASA from aircraft platforms as a proof-of-principle [3,4]. The major obstacle to extract the ocean color information is the contribution from the Earth’s atmosphere, which must be subtracted from the sensor signal. The oceanographic information is approximately only 5 % to 10 % of the detectable signal and as such, it presented a challenge to calibrate the sensor and assess the accuracy of its measurements.

The requirement of adapting laboratory standards for remote sensing measurements at the NBS was at its infancy during the beginning of this period. Ralph Stair, William (Bill) Schneider, William Fussell and Henry Kostkowski and co-workers at the NBS received partial funding from NASA to develop radiometric standards for calibrations of satellite sensors. They established a tungsten halogen lamp and a tungsten strip lamp as field-deployable irradiance and radiance secondary standards traceable to blackbodies at NBS. The uncertainties in the radiometric output were in the 1 % range for these transfer standards. Their work and that of other leaders in the field at that time were summarized in Ref. [5].

As Warren Hovis and John Knoll put it in Ref. [4], “calibration of the solar reflectance bands presented a problem in that none of the calibration sources available from the NBS, either the radiance or irradiance standards, could begin to fill the 10-cm aperture (of the sensor optics) in a way that simulated an extended source, such as earth.” They tried various approaches and finally decided to use an internally illuminated large diameter integrating sphere with multiple lamps to produce diffuse radiance at the exit port with the diameter required to calibrate the large aperture sensors. The sphere they used at the Goddard Space Flight Center (NASA) for the CZCS calibration was of 76.2 cm diameter, and its circular aperture was 29.8 cm. They used a NBS quartz iodine irradiance standard for calibrating a smaller sphere which in turn was used to calibrate the large sphere, thereby establishing traceability to the international radiometric scale (SI) realized at the NBS. The procedure was adopted by other laboratories, and integrating spheres became the main sources of satellite sensor calibration for solar reflective bands.

Warren Hovis actively led a program at NESS Satellite Experiment Laboratory to develop high efficiency integrating spheres. According to one of us (Weinreb), “The laboratory was overrun with integrating spheres, some big, some small, in various stages of development with white paints coating their inside walls. The place had a reputation throughout NESS for looking as if it housed a family of shmoos.” (A shmoo is a friendly, placid, milk-and-eggs-supplying creature dreamt up by cartoonist Al Capp – the inventor of “Li’l Abner.”) The significance of establishing a white diffuse material with a flat response across the solar reflective region also became an important area of research at NBS during this period. Various grades of barium sulfate were found to satisfy the primary requirement of stability of diffuse reflectance over time under ambient conditions. The inside of the Goddard sphere was painted with barium sulfate. Drawing from the reports of Frank Grum of Eastman Kodak and Max Saltzman of University of Los Angeles (UCLA) of a new superior white material, NBS researchers Jack Hsia and Vic Weidner thoroughly characterized the material known as PTFE (polytetrafluoroethylene) [6]. It is also known as Dupont’s Teflon^®^ powder and Allied’s Halon G-80™. The research at NBS showed that superior reflectance properties are exhibited when PTFE powder was sintered and packed. Based on NBS’s research findings, Art Springsteen at Labsphere, Inc., produced the commercial grade of the sintered PTFE called Spectralon™, which became the material of choice for reflectance measurement instrumentation around the world [7].

**Fig. 2:**
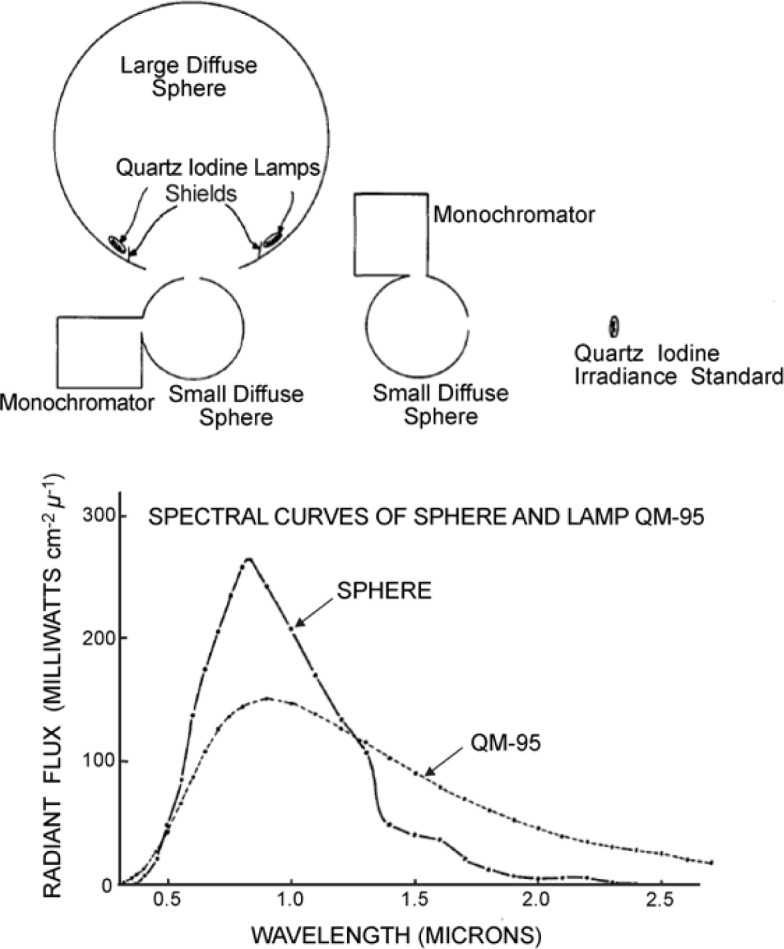
Integrating sphere calibration scheme (Ref. [4]).

Hovis and Knoll [4] brought into operation a well-characterized 180 cm diameter integrating sphere at NOAA for calibrating sensors. The calibration procedure is schematically shown in the upper part of Fig. 2, establishing traceability to the NBS irradiance scale by the quartz iodine irradiance reference standard from NBS nominally known as QM-95. The procedure was accomplished by placing the QM-95 at the distance specified by the NBS for known irradiance at the aperture of a small diffuse sphere attached rigidly to a monochromator. As a first step, the small sphere with its monochromator was calibrated for the QM-95 irradiance at its aperture. The small sphere with its attached monochromator was rotated so that the small sphere aperture was tangential to the aperture of the large sphere as shown in Fig. 2. The large sphere was illuminated by the quartz iodine lamps inside, located symmetrically in a circle with diameter larger than the aperture of the sphere, and shielded from direct view by metal shields coated with white diffusing paint. The large sphere is calibrated for its radiance by knowing the distance between apertures of the two spheres in the set up and by comparing the monochromator output when viewing the QM-95 and the large sphere as a function of wavelength. The spectral radiance from the large sphere is shown in comparison with QM-95 in the bottom part of Fig. 2.

The set up was used for calibrating sensors to be flown on aerial platforms or launched into space on satellites. The aerial platform sensors are flown as under-flights to the sensor on orbit for simultaneously measuring and comparing the reflectance of potential calibration sites on the earth. The use of potential known targets for calibrating the sensor is called vicarious calibration. The pre-launch calibration of sensors need to be validated post-launch to achieve accurate radiometric field measurements from space as the sensor characteristics change due to the stress of the launching process and continued exposure to the space environment [8]. The use of vicarious calibration is discussed further in Sec. 2.2 for the case of AVHRR sensor.

### 2.2 The AVHRR

The sensor for whose calibration NOAA sought guidance from NIST in the early period was the AVHRR, which was used operationally in the POES program. The vendor for the AVHRR, ITT, followed the procedures of Hovis et al. [4], which employed an integrating sphere and NBS standard irradiance lamps for pre-launch calibration. The two AVHRR channels in the solar-reflective portion of the spectrum presented a challenge for on-orbit calibration, as they are designed with no on-board calibration instrumentation and depend entirely on vicarious calibration methods with ground targets for validation of their pre-launch calibration. Peter Abel of NESDIS, who had considerable experience with atmospheric sounding and sensor calibration, performed the technical oversight for the pre-launch and post-launch calibration effort. He relied on NIST for much of the work to ensure that proper practices were used in the calibration and to validate claims of traceability to NIST standards. Abel and his team conducted aircraft experiments to measure the reflectance of potential Earth vicarious calibration targets such as White Sands, NM for the two solar-reflective channels of the AVHRR [9,10]. Their experiments allowed them to track the changes in the responsivity of the solar reflective channels of the NOAA-9 AVHRR from their pre-launch values. Using Hovis’s optical instrumentation on the U2 Lear Aircraft [11], they obtained aircraft measurements from an altitude of 18 km simultaneous with and along the same view to see the same target area as the satellite observations over White Sands. They selected White Sands because the reflected solar radiation from its gypsum dunes make it one of the brightest surface targets available, allowing it to cover the upper dynamic range of the AVHRR channels. In addition, the area is relatively cloud free and has a relatively stable atmosphere [12]. Their results showed an essentially indistinguishable change from pre-flight values of +2 % for channel 1 (570 nm–700 nm) and −2 % for channel 2 (710 nm–1000 nm) 257 days after launch [9,10]. However, their U2 measurements compared with NOAA-9 observations after 690 days in orbit showed a degradation of 12 % for channel 1 and 19 % for channel 2. The pre-launch values were measured 1750 days (~5 years) before launch and the estimated overall uncertainty of the above-quoted results was ±5 % [10]. Upon the invitation of Abel at NESDIS in August 1989, NIST researchers reviewed the pre-launch calibration process and tentatively concluded the current absolute calibration of the AVHRR is no better than ±5 %. They recommended procedures to improve the uncertainty to approach the 1 % level primarily using standard detectors from NIST [13].

In August 1990, Abel left NESDIS and was succeeded on the AVHRR work by Nagaraja Rao, an expert on atmospheric radiation. Earlier, as a contractor to NESDIS, Rao had written NOAA Technical report NESDIS 36 [14], which became the definitive description of the pre-launch calibration of solar reflective channels (channels 1 and 2) of the AVHRR.

The thermal channels of the AVHRR (nominally 3.55 μm–3.93 μm; 10.3 μm–11.3 μm; and 11.5 μm–12.5 μm) are calibrated on orbit from views of cold space and a blackbody in the instrument itself. Before launch, the calibration was performed by the instrument vendor, ITT, in their thermal vacuum chamber with a honeycomb-cavity laboratory blackbody equipped with platinum resistance thermometers whose temperature calibration is traceable to NBS. They claimed ±0.35 K absolute uncertainty for the calibration of the laboratory blackbody [15], based on models that include the thermal gradients and the emissivity of the black paint coating of its surfaces. The AVHRR’s internal blackbody is also a honeycomb cavity array, and it is maintained at the sensor housing temperature. The uncertainty of its calibration was estimated at 0.4 K [15]. The overall uncertainty of the sensor calibration in orbit was estimated to be ±0.55 K [16].

## 3. Legacy of Recent Interactions

### 3.1 Developments at NOAA

**Fig. 3:**
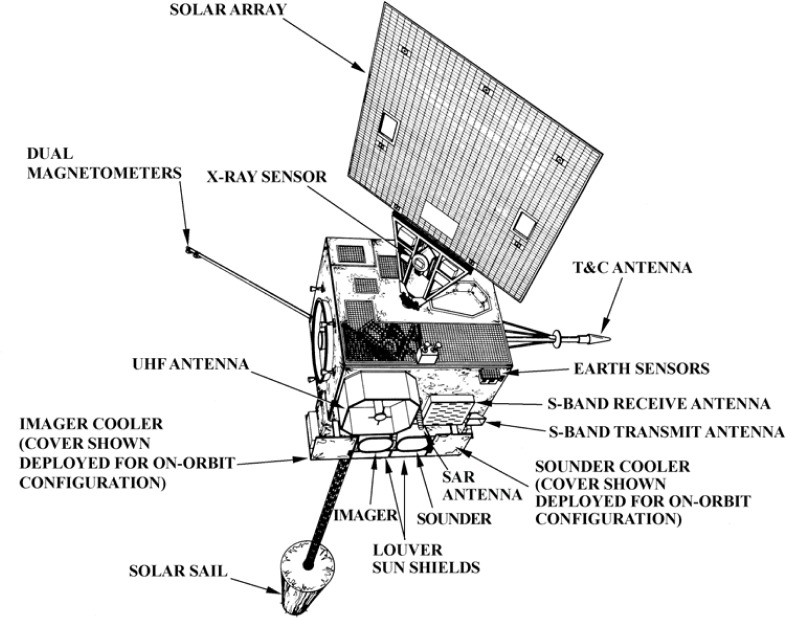
GOES I-M satellite sensor configuration (source: “Weather Satellites” American Meteorological Society, Boston 1990 [2]).

The other satellites that NASA developed for weather monitoring with NOAA funding were the Geostationary Operational Environmental Satellites (GOES). The initial GOES series were spin stabilized and viewed the earth only 10 % of the time. The development of the GOES I-M series, the first three-axis stabilized geostationary satellites to view the earth 100 % of the time, began in the mid-1980s, directed by NASA/GSFC and funded by NOAA. The first of the new series was launched on April 13, 1994, and was renamed GOES-8 on achieving geostationary orbit. The GOES satellite sensor configuration is shown in Fig. 3.

The primary instruments on the GOES I-M satellite are the Imager and the Sounder. The Imager and Sounder are manufactured by ITT Industries, Inc. The Imager is a 5 channel scanning radiometer with one visible (550 nm to 750 nm), one shortwave (3.80 μm to 4.00 μm), two midwave (6.50 μm to 7.00 μm; 10.20 μm to 11.20 μm) and one longwave (11.50 μm to 12.50 μm) infrared channels. It senses radiant energy and reflected solar energy from the Earth and produces visible and infrared images of earth’s surface, oceans, cloud cover, hurricanes and severe storms. The Sounder is also a scanning radiometer featuring nineteen spectral channels (seven longwave, five midwave, six shortwave, and one visible) that yield the sounding products of vertical atmospheric temperature and moisture profiles, surface and cloud top temperatures. The other instruments monitor the space environment (SEM) and provide data on solar flares, solar wind and high energy particle storms that affect space navigation, communication and electrical power transmission. The SEM data helps industries (electrical power distribution, oceanic and aero-space navigation and communications etc.) to modify their operations as needed and mitigate the effects of Coronal Mass Ejections (CME) from the Sun reaching the earth causing geomagnetic storms.

NOAA began interacting with NIST for resolving certain calibration anomalies in the GOES observations. Before the launch of each of these satellites, its Imager and Sounder were thoroughly tested by ITT, their manufacturer. These tests were carried out in the 1990s, beginning with GOES-I in 1993. They included calibration and characterization of the thermal-infrared channels, performed in a thermal-vacuum chamber. One of the puzzling results of the calibrations was that, for each instrument, the brightness temperature of its internal calibration target (ICT), determined radiometrically by the instrument itself after it had been calibrated against ITT’s laboratory blackbody, differed from its temperature as indicated by the PRTs embedded in it. These differences, termed “ICT errors,” were of the order of 0.5 K to 1.0 K and varied over both channel (i.e., wavelength) and the instrument’s operating temperatures. This was not the first appearance of ICT errors. They were also observed earlier [14] in the pre-launch calibrations of the infrared channels of the AVHRR’s on the polar-orbiting NOAA-9, -10, and -11 satellites. In addition, the response (counts out versus radiance in) in the shortwave infrared channels was observed to be non-linear, despite theoretical predictions and other evidence that it should be linear.

A sufficient (but not necessary) hypothesis that could explain the ICT errors and the shortwave non-linearity was offered by Edward Wack of MIT Lincoln Laboratory in 1996 [17]. He suggested that the radiances of the laboratory blackbody used in the calibration were erroneous. These radiances had been traced to NIST standards not by any radiometric measurements but only through the calibration of the PRT temperature sensors embedded in the blackbody. The radiances were inferred by calculation from the PRT measurements with the assumption that the blackbody’s emissivity was essentially unity. Earlier work by H. Farthing at NASA [18] had shown that if ITT’s laboratory blackbody had temperature gradients between its radiating surfaces and the locations of its embedded PRTs, then the computed blackbody radiances would be incorrect, and the consequence in the calibration would be both an ICT error that varied with instrument temperature, and errors in the magnitudes of the derived non-linearities in every channel. This, however, could not explain the additional observation that the ICT error varied with channel (wavelength). Wack recognized that this could be explained by the wavelength dependence of the Planck function in a situation where the emissivity of the laboratory blackbody is less than that of the ICT. In summary, the ICT and non-linearity errors could be explained by a combination of two hypothesized deficiencies of the laboratory blackbody—an emissivity less than that of the ICT and the presence of temperature gradient between its PRTs and its radiating surfaces. In 1997, one of us (Weinreb) of the NESDIS Office of Research and Applications (ORA) gave talks [19,20] on these problems at two conferences, which drew the attention of Al Parr, the division chief of the then NIST Optical Technology Division (OTD). He pointed out to NOAA the NIST capabilities in those areas to support NEDIS’s satellite-related calibration activities.

In May 1998, yet another problem in GOES calibration surfaced, giving NESDIS/ORA additional motivation to seek cooperation from NIST—in this case to validate the spectral response functions of the GOES instruments. In early 1998 it was noticed that the simultaneous brightness-temperature observations of the same scenes on the Earth by the Sounders on GOES-8 and GOES-10 disagreed by up to 5 K. Later that year it was also found that there was poor agreement between the measured brightness temperatures in those channels and the brightness temperatures calculated – using spectral response functions provided by the filter vendor (OCLI) – from in-situ temperature and water-vapor profiles. These discrepancies threw suspicion on the accuracy of the spectral response functions provided by the GOES filter vendor. The GOES Program approved the recommendation of the NESDIS ORA to subject some of the witness samples of these filters to the NIST measurement process.

### 3.2 NBS Becomes NIST

The NBS was reorganized in the late 1980s to form the NIST through Congressional legislation to emphasize technology development and standards to forge US innovation and industrial competence. Under the reorganization, NIST expanded the radiometry and optical characterization facilities. NIST developed the High Accuracy Cryogenic Radiometer (HACR) as the NIST primary standard for optical power with 0.01 % uncertainties on par with an international effort to reduce uncertainties in radiometric calibrations of optical radiation detectors [21]. The HACR is now replaced with an improved version at NIST with modern electronics, called the Primary Optical Watt Radiometer (POWR). Supplementing NIST’s internal support for radiometric calibration activities, the Sea-viewing Wide Field-of-view Sensor (SeaWiFS) and the Earth Observing System (EOS) projects at NASA and the Marine Optical Buoy (MOBY) project at NOAA/NESDIS funded NIST to build a number of transfer standard radiometers [22,23] and carry out various calibration and validation activities [24]. Another development during this period was the receipt of DoD funding, which enabled NIST, led by Keith Lykke, to acquire tunable lasers to illuminate an integrating sphere to provide radiance and irradiance calibrations. In this approach, tunable lasers are coupled to an integrating sphere to produce either uniform irradiance at a reference plane or uniform radiance within the sphere exit port. Using this source, detectors or radiometers are calibrated directly against NIST reference standard detectors. This capability was called Spectral Irradiance and Radiance Calibration using Uniform Sources (SIRCUS). It became the key capability for detector-based calibrations at NIST with an uncertainty at the 0.1 % level [25]. DoD also supported the development of the water bath blackbody and various fixed-point blackbodies to provide source-based radiometric scales.

**Fig. 4:**
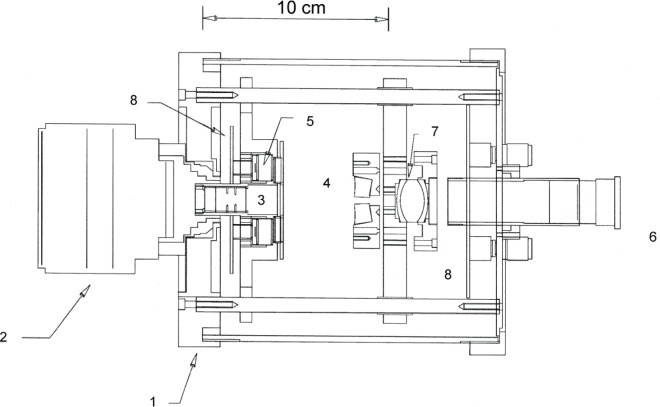
SXR schematic layout (Ref. [24]); 1) mounting point to install the SXR to view the radiance source; 2) 85 mm focal length objective lens; 3) precision field stop aperture; 4) six, wedge-shaped fold mirrors; 5) six interference filter and detector assemblies; 6) optical alignment eyepiece; 7) optical alignment relay lens; 8) electronic circuit boards.

The first transfer standard radiometer at NIST was developed by one of us (Carol Johnson) in the 1990s to transfer SI-traceable radiance scales to the SeaWiFS project at NASA. It is called the SeaWiFS Transfer Radiometer (SXR) [26]. NASA developed and operated the SeaWiFS in space for ocean color measurements from 1997 to 2010. The SXR was a portable six channel imaging radiometer with six interference filters at 411.2 nm, 441.5 nm, 486.9 nm, 547.9 nm, 661.7 nm, and 774.8 nm each with a nominal band pass of 10 nm. Its schematic optical setup is shown in Fig. 4. It provided SI-traceable detector-based spectral radiance validation of the integrating sphere sources and illuminated diffuse plaques that are used for inter-comparison and pre-launch calibration of the SeaWiFS instrument. The relative combined standard uncertainty of spectral radiance measurements with the SXR was between 0.6% and 1.3 % accounting for channel-to-channel variation. Based on the success of the SXR, NIST developed the Visible Transfer Radiometer (VXR) for the EOS program. The SXR design is essentially implemented in the VXR with improved field-of-view response and further extension into the infrared by replacing the filter at 441 nm with a filter at 870 nm. The VXR has been characterized and calibrated using different facilities at NIST to a relative combined standard uncertainty of less than 1 % [27]. It is available from NIST as the SI-traceable transfer radiometer for deployment at customer facilities to provide radiance characterization and validation of integrating sphere sources. Its application to GOES-R Advanced Baseline Imager (ABI) pre-launch characterization is discussed in Sec 3.4.4.

NOAA/NESDIS and NIST also collaborated to provide SI-traceable radiance and irradiance calibrations for the MOBY program developed by NOAA and NASA for obtaining ground “truth” for vicarious calibration of ocean color satellite sensors, SeaWiFS and MODIS. As discussed in Sec. 2.1., for the case of the CZCS sensor, the removal of atmospheric radiance is an essential part of ocean color measurements and as such the instruments in space are vicariously calibrated. The MOBY deployed near Hawaii in the Pacific uses the Marine Optical System (MOS) fiber-coupled to sensor fore-optics to measure the upwelling radiances and down welling irradiances within the ocean [28]. The high resolution spectral data of upwelling radiance are convolved in real time with the satellite band spectral response functions to provide band-averaged water-leaving spectral radiances for vicarious calibration of the satellite sensor. NIST characterized MOBY spectrographs using SIRCUS lasers and developed a stray light correction of the spectrographs [29]. NIST also developed single-channel narrow-band filter radiometers called Standard Lamp Monitors (SLM) for SI-traceable calibration maintenance of the MOBY reference sources. The SI-traceable uncertainty of the radiances of MOBY reference sources is estimated to be 3 % (k = 1); independent NIST measurements have validated the result [30].

**Fig. 5:**
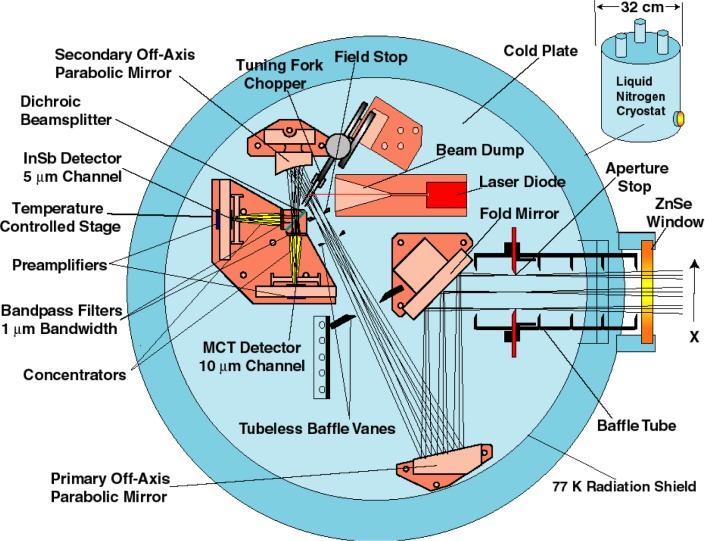
TXR optical layout (Ref. [29]).

To transfer the radiometric scale in the infrared from NIST to customer facilities, one of us at NIST (Joe Rice) developed the Thermal-infrared Transfer Radiometer (TXR) under funding from NASA’s EOS project. The TXR, shown in Fig. 5, is a dual-channel filter radiometer with 1 μm bandwidth channels at 5 μm and 10 μm. It is made available to test customer blackbody emissivity and calibrate the contact thermometers in terms of the radiance temperature [31]. The NASA EOS Project also funded NIST to build a cryogenic chamber at 80 K background called the Medium Background Infrared (MBIR) chamber. The TXR was calibrated in the MBIR chamber using a very high emissivity cavity type (ε = 0.9997) NIST cryogenic blackbody standard. The TXR design allowed it to be operated in ambient as well as in vacuum. Its calibration was validated with the NIST water bath blackbody standard. The TXR calibration was maintained in the field with a stable blackbody that is carried along with TXR during deployments.

For infrared calibrations beyond 2 μm, DoD support enabled NIST to build the Low Background Infrared (LBIR) facility, consisting of two cryogenic chambers and two absolute cryogenic radiometers, for the calibrations of cryogenic blackbodies from facilities of DoD and its missile defense contractors [31,32]. In building these capabilities, the scientists at the Optical Technology Division of NIST (OTD) became experts by the late 1990s in designing and building cryogenic radiometers at NIST. In addition, they developed unique capabilities for characterizing infrared optics by measuring optical properties such as specular and diffuse reflectance and transmittance of bulk materials in the 2 μm to 18 μm wavelength range [33,34] as a function of temperature from 4 K to ambient, with the goal of meeting the requirements for higher sensitivity and higher accuracy for missile defense applications.

### 3.3 Start of Formal NOAA/NIST Cooperation

The formal procurement process within NOAA to seek cooperation from NIST began in July 1999 under the technical direction of one of us (Weinreb), who was the GOES calibration scientist at the NESDIS Office of Research and Applications. He wrote a requirements document that began with the truism that accurate calibration is needed if GOES is to provide atmospheric observations that satisfy its users’ stated uncertainty requirements. It alluded to previously observed inconsistencies in NOAA’s satellite measurement from GOES and POES satellites, attributing them to inadequacies in calibration. It mentioned the specific difficulties, described above, in the pre-launch calibration of the GOES I-M instruments. It concluded with the recommendation for the establishment of a long-term collaboration with NIST for support of pre-launch calibration, beginning with TXR measurements of ITT’s laboratory blackbody.

On August 12, 1999, NIST responded with a proposal that offered a program of continuing support for calibration of NESDIS’s POES as well as GOES sensors. The goal would be to reduce calibration uncertainties to 0.1 K in radiance temperature. The planned work would be the TXR measurements of ITT’s laboratory blackbody for the GOES Imagers. But the program would also include such other tasks as end-to-end reviews of the calibration procedures of the instrument vendors, measurements of the spectral response functions of the GOES instruments’ bandpass filters, participation on NESDIS calibration panels, and collaboration with NESDIS in developing a long-term program of pre-launch calibration support. These tasks were modeled upon ongoing efforts for NASA EOS and DoD projects.

The first measurements made by NIST for NOAA were interferometer measurements of the spectral response functions of witness bandpass filters for selected channels of the GOES-8 and -10 Imagers and Sounders. These measurements were carried out by Simon Kaplan of NIST in March and April 2001 [35]. In some channels, the NIST measurements agreed with the spectral response functions provided by the filter vendor, but in several others, that was not the case. In those channels the NIST spectral response functions resolved the problems that were seen with the GOES-8 and -10 observations. In particular, closer agreement was obtained between calculated radiances and the observations when the NIST spectral response functions were substituted for those from the filter vendor in the calculations. These findings provided a strong justification for employing NIST to validate the spectral response functions of NOAA’s satellite radiometers. (It should be mentioned here that ITT is not convinced that the NIST measurements of the witness filters say anything about the filters on the flight instrument itself. They argue that there can be considerable variations in spectral response between witness samples and the flight filters, and that these variations could be as large as or larger than the difference between the measurements by the filter vendor and NIST. Nevertheless, because the NIST measurements are more consistent with the observations, NOAA is convinced that the NIST measurements are superior.)

The TXR measurements of the radiances and emissivities of ITT’s laboratory blackbody were performed by one of us (Joe Rice) of NIST during a two-week period in July 2001 using the NIST TXR. The laboratory blackbody at ITT, called External Calibration Target (ECT), had been in regular usage at ITT for the pre-launch calibration of operational GOES imagers. The TXR data analysis was structured to infer an (non-unity) emissivity and any temperature gradients in ITT’s laboratory blackbody that would allow the radiances of the blackbody computed from its temperature sensors to be in accordance with the TXR radiance observations. The analysis also produced corrections to each temperature of the laboratory blackbody, which could be used with an emissivity of unity to calculate corrected radiances [36]. These corrections agreed qualitatively with the empirical corrections derived earlier by Wack [37]. NIST help resolved the issues identified: ICT errors and short wave non-linearity, and corrections to ECT temperature sensor readings. This work provided strong justification for the use of NIST to validate the radiance scales of the laboratory calibration targets on which the calibrations of NOAA’s satellite radiometers are based.

### 3.4 NIST – NOAA/NESDIS ORA (Later the Center for Satellite Applications and Research [STAR]) Collaboration to Improve Operational Satellite Data Accuracy

#### 3.4.1 NOAA Supports TXR Trip to Miami

NOAA participated in funding the Committee on Earth Observing Satellites (CEOS) intercomparison of blackbodies used to calibrate radiometers deployed on ships to measure sea surface temperature. The NASA EOS Project also sponsored the event. NIST participated in the comparisons held at the University of Miami’s Rosenstiel School of Marine and Atmospheric Science (RSMAS) in 2001. NIST employed the TXR for this purpose; consequently, this study provided an independent experimental check of the SI-traceability of sea surface temperature measurements [38]. The NIST TXR was employed in reasonably controlled laboratory conditions to view several cavity blackbodies and measure the brightness temperatures of each. The laboratory blackbodies were five in total, one the NIST water bath blackbody (WBBB), which served as the reference blackbody, and four other participating blackbodies (BB): The RSMAS BB, the Jet Propulsion Laboratory (JPL) BB, the Combined Action for the Study of the Ocean Thermal Skin (CASOTS) Rutherford Appleton Laboratory (RAL) BB, and the CASOTS Southampton Oceanography Centre (SOC) BB. All of these were operated independently of each other in the same laboratory at RSMAS during the workshop. Each BB had a calibrated thermometer which was used to determine the temperature of the cavity. Careful use of these blackbody targets, whose calibration was now referred to the TXR standard, to calibrate seven ship-based radiometers that participated in the workshop, including the Marine-Atmosphere Emitted Radiance Interferometer (M-AERI) used in the validation of satellite-derived skin sea-surface temperatures, therefore resulted in validation data sets that had uncertainties within ±0.1 °C [39]. This intercomparison demonstrated some of the verification capabilities that are now available to the environmental remote sensing community with the use of the NIST TXR.

#### 3.4.2 NIST Measurements of HIRS Bandpass Filters

Following the successful NIST measurements of GOES filters mentioned earlier, NESDIS employed NIST to measure the spectral response functions (SRF) of the HIRS (High-Resolution Infra-Red Sounder) instruments on the POES series. Since the beginning of the POES series in 1978, radiances observed in some HIRS channels differed significantly from the radiance values produced by forward calculations based on coincident measurements of atmospheric temperature and humidity profiles. These differences, similar to the ones affecting the GOES Sounders, led such data users as the National Weather Service to apply large bias corrections to the measured radiances before assimilating them into its forecast models. The question was, “Could the differences be the result of incorrect knowledge of the spectral response functions used in the forward calculations?” In 2001, one of us (Weinreb) proposed that NIST verify the spectral response functions of the HIRS’s bandpass filters. Funded by the NESDIS Office of System Development, Simon Kaplan of NIST measured the spectral response functions of witness samples of the bandpass filters for a HIRS instrument [40]. The measurements showed a pattern of differences from the vendor-provided SRFs that were consistent with some (but not all) of the discrepancies between observation and calculation encountered in orbit [41].

#### 3.4.3 NIST Measurements of GOES-13 Bandpass Filters

In March 2007, NOAA scientists Timothy Schmit and Mathew Gunshor observed a 2.4 K cold bias, relative to observations with well-calibrated instruments on other satellites, in the observations from channel 6 (13.3 μm) of the GOES-12 and -13 Imagers [42]. They hypothesized that the bias could be the result of an error in the filter-vendor-provided SRF, and that a linear shift in the spectral response of a few wavenumbers could eliminate it. Later, Wu et al. [43] presented results for GOES-13 that strongly supported that hypothesis and quantified the required shift in the SRF. Based on previous successes with NIST measurements of filter spectral response functions for the GOES instruments and the HIRS, the NESDIS Center for Satellite Applications and Research (STAR) decided to have NIST measure the spectral response functions of witness samples of this channel. Witness samples for channel 6 of the GOES-12, -13, and -14 Imagers were shipped to NIST in the summer of 2009. Simon Kaplan made the measurements and issued his report on January 28, 2010 [44]. An analysis comparing the calculated radiances based on the NIST results and the vendor-provided SRFs is currently in progress.

#### 3.4.4 GOES-R

GOES-R (Geostationary Operational Environmental Satellite-R Series) is the next generation of geostationary weather satellites. The first satellite with its sensors is scheduled to be launched in 2015. The ABI is the primary sensor on GOES-R for imaging Earth’s weather, climate and environment with advanced capabilities compared to the previous GOES imagers. It has 16 different spectral bands spanning the visible to thermal infrared and will provide more spectral, spatial and temporal coverage than the current GOES system. A workshop was held on October 20, 2003, at NIST with the participation of researchers from NASA (Sandra Cauffman, Joe Criscone, and Dennis Chesters), NIST (Carol Johnson, Steve Brown, and Joe Rice) and NOAA (Weinreb) on the services NIST could offer to a satellite remote sensing program such as GOES-R. In January 2004 one of us (Weinreb) retired from NOAA but returned in March 2004 as a part-time contractor, with responsibility to organize the GOES-R calibration activities. That included starting a management-level calibration steering committee, starting a working-level calibration working group, initiating a relationship with NIST to verify the traceability of the GOES-R sensors’ calibration, and acting as liaison with NIST. In December 2004 one of us (Johnson) at NIST wrote a five-year statement of work for NIST support of GOES-R. It contained tasks in two areas: 1) reviewing GOES-R calibration plans and participating on panels and committees, and 2) carrying out measurements to verify the traceability of GOES-R calibration, e.g., the use of VXR and TXR to characterize ABI laboratory calibration sources, measurements of spectral response functions of the ABI, and experiments to assess vendors’ claims of traceability. Later, space weather monitoring instruments on the GOES-R satellite platform were also included by NOAA for traceability verification.

The GOES-R Program at NOAA began funding these activities in fiscal year 2005. The funding was renewed in FY 2006 and FY 2007. It supported work at NIST until FY2009, when a more formal relationship between NESDIS and NIST was established. The thrust of the work before FY2009 consisted of reviewing GOES-R calibration designs and plans, participating in reviews and on committees, and planning the measurements and upgrading equipment and facilities for verifying the radiance scales of the calibration sources of the ABI Vendor, ITT^2^ with, e.g., the TXR and the VXR. The Calibration Working Group and the Steering Committee were staffed and functioning. At the same time, the Department of Commerce mandated that funding of NIST by NESDIS should be covered by a formal Memorandum of Understanding (MOU) between the two institutions. Responsibility for writing the MOU and for the liaison with NIST was taken on by one of us (Cao) at NESDIS/STAR, who had taken over as chairman of the Calibration Working Group (CWG) for GOES-R. The overall goal of the GOES-R MOU is to have NIST provide instrument calibration and characterization services to enable NOAA to verify and document the radiometric metrology developed and maintained by the instrument manufacturers and other scientific participants. Considerable progress has been made by following a time schedule in establishing NIST traceability for the calibration and characterization of the ABI sensor, the Solar Ultraviolet Imager (SUVI), and the EUV and X-Ray Irradiance Sensor (EXIS) instruments. The SUVI and the EXIS sensors observe the Sun from extreme ultraviolet (EUV) to soft X-ray wavelength range and provide data that is referred as space weather. This data provides advanced warning to various government agencies and industry on possible effects on the Earth’s environment and enables them to take preventive steps to avoid serious disruptions of communications, navigation and electrical power supply. The following are some of the specific highlights of current progress on GOES-R based on the MOU.

- The External Calibration Target (ECT) developed by ITT for the pre-launch calibration of the ABI sensor has been tested and validated by NIST for its performance. For this purpose, the NIST TXR was deployed to the ITT cryogenic chamber in Rochester for in situ measurements of the ECT. The TXR was located in the chamber at the same physical location where the ABI sensor was located for its testing. The geometry of the set up was to emulate the conditions of ABI testing and validate the performance of the ECT to meet the requirements. The emissivity of the ECT was measured and the ECT radiance temperature scale was validated [45].
- Two FEL lamps that illuminate the ABI’s solar port for pre-launch testing were validated by NIST for their spectral irradiance scale. These lamps were calibrated at NIST. To establish the on orbit traceability of the radiometric response of the ABI sensor solar reflective channels, the sun is used to illuminate an on-board diffuse reflectance standard that is sub-entrance pupil for ABI. The lamp standard for spectral irradiance (type FEL) serves to illuminate the ABI solar port during pre-flight calibration and validate the spectral irradiance responsivity assigned on orbit using the sun [46].
- The spatial uniformity of FEL-based irradiance scale is tested at NIST to use the FEL at distances other than the nominal 50 cm to enable the use of FEL lamp to fill the field-of-view of the ABI sensor. NIST performed the measurements using the gonio filter radiometer (GOFR) and validated those measurements using the NIST gonio-spectroradiometer facility. NIST provided the test results with quantified uncertainties to help the use of FELs at desired distances for pre-launch testing of ABI [47].
- NIST (Keith Lykke and Steven Brown) performed calibration and characterization studies for end-to-end testing of the ABI Prototype Model (PTM) at ITT/Fort Wayne, IN using the NIST Travelling SIRCUS [48]. The results helped to compare and verify the spectral response functions based on the vendor model for the 470 nm, 640 nm, and 860 nm channels [49].
- The 165 cm diameter large sphere at ITT, Ft. Wayne, IN, which is used to illuminate the ABI earth-port for determination of its visible-near infrared spectral radiance response functions, was measured in April 2013 by NIST (Carol Johnson and Steve Maxwell) to validate its radiance scale. This scale is established using a NIST-calibrated integrating sphere and a scanning monochromator. NIST used the VXR and two spectrophotometers to perform the measurements. A NIST Portable Radiance (NPR) source developed by Steven Brown served as the radiance standard for this validation activity.
- The filters for the 6 witness samples of the VIS-Near IR bands and the 10 witness samples for the IR bands for the ABI sensor have been characterized at NIST at high spectral resolution for various incident angles at specific temperatures meeting the ABI sensor operational requirements. The results were provided with uncertainty budgets to help compare with vendor-provided measurements for the real ABI filters [50].
- The first flight unit of EXIS has been calibrated at the Spectrometer Calibration Beamline (BL-2) of the NIST Synchrotron Ultraviolet Radiation Facility (SURF III). Several more units are planned to be calibrated in the near future. Calibrations already completed for the EXIS project include the engineering test unit, rocket underflight unit, and flight unit of the X-Ray Sensor and the flight unit of the Extreme Ultraviolet Sensor [50].
- NIST staff has been working closely with the SUVI instrument development team in addressing the use of NIST standards to meet the SUVI calibration requirements and in evaluating the metrology done by the vendor. Several calibrations have already been provided at NIST SURF III. The work performed thus far includes 1) Calibration of several transmission gratings using the current BL-7 Beamline in support of end-to-end calibration. 2) Testing of witness samples from the SUVI filter deposition runs by the vendor. The NIST results confirmed the vendor estimates of sample film thickness satisfying the minimum transmission requirements. NIST data provided input on the unexpected large batch to batch variation to be addressed by the vendor [51].

### 3.5 Monitoring Climate Change and Improvement of Weather Prediction Models – NOAA and NIST Cooperation

At the beginning of this century, global climate change became an important issue worldwide, and the absolute accuracy of satellite-based radiometric data of the earth was recognized as a crucial factor in assessing climate change. The three agencies, NASA, NIST, and NOAA, played a pivotal role in bringing the remote sensing community together to address measurement challenges for satellite-based climate observations. Steve Ungar of NASA and one of us (Cao) of NOAA played active roles in CEOS [52] as chairs of the Working Group on Calibration and Validation (WGCV) during the first decade of the century in building strong international cooperation for improving pre-launch and post-launch calibration and validation of sensors. NIST actively participated in CEOS/WGCV activities, promoting the development of high accuracy laboratory radiometric standards and the dissemination of scales through transfer standards traceable to SI units (SI-traceability).

In 1994, NOAA, DoD, and NASA had created an Integrated Program Office (IPO) within NOAA to develop, manage, acquire, and operate the National Polar-orbiting Operational Environmental Satellite System (NPOESS). The NPOESS satellite program was intended to reduce costs and increase efficiency by combining NOAA’s POES and DoD’s Defense Meteorological Satellite Program (DMSP) [53]. The NPOESS program initiated the NPOESS Preparatory Project (NPP) to reduce developmental risk by early flight-testing of instruments, thereby demonstrating and validating the performance of the new NPOESS sensors, including the Visible/Infrared Imager radiometer Suite (VIIRS), an imager, and the Cross-track Infrared Sounder (CrIS), a sounder. The NPOESS/IPO entered into a co-operative research agreement with NIST and sponsored the development of calibration capabilities at NIST to serve NPOESS requirements for the NPP sensors.

Steve Mango, the chief scientist of the NPOESS IPO (1995–2008), promoted the development at NIST of thermal infrared standards and measurement methodologies for high accuracy pre-launch calibration of the NPOESS/NPP Cross-track Infrared Sounder (CrIS) by its contractors ITT and Northrop Grumman. The NIST Optical Technology Division (OTD) received funding from the NPOESS IPO that helped NIST thoroughly characterize the TXR in preparation for field deployment to validate the calibrations of the blackbody targets used by such aerospace contractors as ITT for calibrating remote sensing instruments in the thermal infrared. The successful deployment of the TXR in support of the GOES program, already discussed in Sec. 3, has been an example of the legacy of the cooperation of NOAA and NIST researchers working to resolve operational satellite sensor calibration issues. NOAA is currently sponsoring NIST to calibrate the ECT at ITT, Ft Wayne, IN to support the CrIS FM1 sensor pre-launch calibration for the newly organized Joint Polar Satellite System (JPSS) [54], which is a NPOESS follow up organization for NOAA. Following the retirement of Mango, Bruce Guenther, a NASA retiree and a lead promoter of the NASA EOS/NIST collaboration, became the contract scientist on the sensor segment for leading the pre-launch calibration of the VIIRS sensor on NPOESS/NPP (now called the Suomi National Polar-orbiting Partnership [S-NPP]). He worked with NIST researchers Keith Lykke and Steven Brown to deploy NIST’s Travelling SIRCUS to Ball Aerospace & Technology Corp. (BATC) for end-to-end testing of the VIIRS solar band channels relative spectral response (RSR) prior to the S-NPP launch in 2011 [55].

Mango also tasked NIST to determine accuracy requirements for instruments in space to monitor climate change. Sponsored by NPOESS IPO, the NIST OTD collaborated with George Ohring of NOAA and James Butler of NASA to organize the joint workshop, “Satellite Instrument Calibration for Measuring Global Climate Change,” in 2002. This workshop was a grand success, with the active participation of nearly 100 scientists and engineers from the three agencies and major NASA aerospace contractors. The workshop addressed the measurement challenges and instrument calibration needs for the accurate space-based measurement of global climate-change variables such as atmospheric temperature and solar irradiance. These variables are critical for assessing the magnitude of, and determining an effective policy response to, global climate change. The workshop NIST report, NISTIR 7047 [56], summarized the required absolute accuracies and stabilities of climate-change variable determinations for use in monitoring climate and verifying predictive climate-change models. It then related the required climate-change variable accuracies and stabilities to satellite-sensor measurement accuracies and stabilities. The report also assessed the ability of present satellite technology to deliver the required measurement accuracy and stability. Guiding principles to maintain and improve the accuracy of satellite remote-sensing measurements through better instrument calibration and validation are also provided in the report.

As a follow up, the second workshop, “Achieving Satellite Instrument Calibration for Climate Change (ASIC^3^),” sponsored by NOAA, NASA, NIST, NPOESS, and the Space Dynamics Laboratory of the Utah State University was organized in 2007. Its goal was to formulate a national roadmap for developing the calibration systems meeting the requirements identified in the first workshop to monitor long-term global climate change. Two overarching recommendations emerged from the workshop [57]. The first recommendation was to conduct a set of satellite benchmark missions to create irrefutable records and calibrate other satellite sensors. Specifically it called for a suite of climate benchmark instruments whose accuracy, through traceability to international standards, can be proven on-orbit. The second recommendation called for a U.S. interagency National Center for Calibration (NCC). It is based upon the realization that the recommendations of the workshop can be implemented only through an integrated national effort in instrument calibration involving the U.S. agencies that launch and/or operate satellites that remotely sense the Earth’s atmosphere and surface – NOAA, NASA, United States Geological Survey (USGS), DoD and Department of Energy (DOE) – and the agency for measurement standards – NIST. The first recommendation concurred with the recommendation in the National Research Council (NRC) Decadal Survey for Earth Science and Applications from Space: National Imperatives for the Next Decade and Beyond, which called for a satellite with benchmark instruments called the Climate Absolute Radiance and Refractivity Observatory (CLARREO). The CLARREO project is currently in the study phase at NASA. Based on the second recommendation, NOAA took the first step and established the NOAA National Calibration Center (NCC) at NESDIS/STAR in 2011 to “facilitate improved accuracy of NOAA’s weather, climate, and ocean models through sharing of technical practices for fine tuning remotely-sensed data from environmental satellites among different programs and agencies. The NCC’s mission is to provide common standards and methodology for the user community as well as encourage communication though a centralized Calibration Knowledge Base. This practice provides support to NOAA’s satellite programs by enforcing stricter and more widespread quality control on satellite data from the Global Earth Observation System of Systems (GEOSS), which will improve efficiency and reduce costs as the community strives to meet the growing needs for high quality satellite data [58].”

Another important development for on-orbit calibration came in the early part of this century from the researchers at NOAA/NESDIS/STAR, (Cao and Andrew Heidinger), who first showed that Simultaneous Nadir Overpasses (SNOs) can be utilized for the satellite-to-satellite intercalibration of radiometers [59]. They with their colleague Jerry Sullivan published the methodology for comparing the AVHRR observations (on POES meteorological satellites) with those from MODIS (on EOS satellites) [60]. This work paved the way for the establishment of the Global Space-based Inter-Calibration System (GSICS) by the World Meteorological Organization (WMO) [61]. Most of the satellite operating agencies around the world joined the GSICS organization and started working on intercalibrations of their satellites. The GSICS executive panel invited the NIST OTD to be a member. NIST was tasked to generate the best practice guidelines for pre-launch calibration of optical sensors that would serve as a common best practice guide for all its members around the world. The NIST OTD released its report in 2009 [48].

Also in 2009, NIST, NOAA, and NASA held a workshop at NIST to develop strategies on how to bridge a possible gap in satellite data for climate in case of a launch delay or other failure in maintaining continuous time series of observations from space. The workshop report [62] was published in 2011. Its recommendations emphasized the importance of having all satellite sensors undergo SI-traceable radiometric calibrations in the pre-launch phase and carry SI-traceable radiometric standards to achieve the highest accuracy measurements in space. The need to calibrate the moon as a calibration target beyond the accuracy of the USGS Robotic Lunar Observatory (ROLO) model [63] was emphasized. The recommendation was to perform SI-traceable observations of the moon from mountain tops and high altitude balloons to minimize atmospheric absorption and contributions from aerosols, and to use NIST-calibrated instrumentation to achieve high accuracy. The goal will make the moon an absolute SI-traceable celestial radiometric standard permanently available for on orbit calibrations and intercomparisons of satellite solar reflective channels of sensors. Any gaps in satellite data in the solar reflective region can potentially be bridged using the moon as an SI-traceable standard. The GSICS model of building a robust intercomparison network of all sensors in space by the SNO technique is highly recommended as a means to bridge the data gaps. The community also recommended that SI-traceable airborne sensor campaigns and SI-traceable radiosonde measurements should be promoted to validate satellite observations for climate, as they would also help mitigate the gaps in satellite data. To enable implementation of the recommendations NIST has built an easily deployable travelling SIRCUS to provide SI-traceable system-level radiance and irradiance calibrations at customer sites. NIST proposed the Lunar Spectral Irradiance and radiance (LUSI) project to radiometrically characterize the Moon from mountain tops and high altitude balloons as recommended in the workshop for use as an SI-traceable on-orbit reference standard for satellites with the required accuracy [64,65].

## 4. Summary

The cooperation between NOAA and NIST scientists to increase the accuracy of satellite optical sensor data to improve the prediction of weather and the monitoring of climate change started at the inception of weather satellites and is on-going. Some of the highlights of this article are summarized here:

- Scientists at the NOAA’s NESDIS began seeking help from NIST with calibration of their laboratory standards from the start of their environmental satellite program. The initial collaborations between the two agencies were confined to the calibration of visible and near-infrared sensors.
- NIST developed standard lamps early-on that helped NOAA and NASA scientists to develop a pre-launch calibration strategy for visible and near-infrared channels of satellite sensors using lamp illuminated integrating spheres. It has become a standard method around the world.
- Also early-on, NIST (formerly NBS) characterized the sintered and packed polytetrafluoroethylene (PTFE) as a diffuse reflectance standard that is now widely used to provide reflected solar radiation for the on-orbit calibration of visible channels. The material is called Spectralon™ by its vendor, Labsphere, Inc.
- In the 1990’s and early 2000’s, NOAA sought and secured assistance from NIST to help diagnose the causes of issues in the performance of the infrared sensors on NOAA’s polar and geostationary meteorological satellites. This cooperation paved the way in 2009 for the generation of a formal Memorandum of Understanding (MOU) between NOAA and NIST for NIST support of the pre-launch calibration in the GOES-R Program. Under this MOU, NIST is validating the calibration of the laboratory calibration standards of GOES-R instrument vendors and the spectral response functions of the GOES-R ABI.
- NIST and NOAA convened workshops, with the active participation of NASA and industrial and academic institutions, that identified the minimum measurement requirements for detecting and monitoring global climate change, the corresponding instrument capabilities needed to make those measurements, and a road-map for achieving the required capabilities.
- The NOAA National Calibration Center (NCC) was established to promote best practices and compile a knowledge base for improving the calibration of sensors whose observations are needed to help improve weather prediction and the monitoring of climate change.
- In a workshop organized by NIST with active participation of NOAA and NASA scientists, calibration strategies were recommended to help bridge gaps in time series of satellite sensor data resulting from launch failures or other operational failures. Responding to the recommendations NIST has built a travelling SIRCUS that is easily deployable at customer sites. NIST proposed the LUSI project to radiometrically characterize the Moon as SI-traceable for use as an on-orbit reference standard for satellites with the required accuracy.

## Footnotes

2 ITT has reorganized and since 2011, it is called ITT Exelis which has the GOES-R ABI contract.

## 5. Acronyms and Abbreviations

ABI: Advanced Baseline Imager (GOES-R)
AMSU: Advanced Microwave Sounder
ASIC3: Achieving Satellite Instrument Calibration for Climate Change
ATN: Advanced TIROS-N
AVHRR: Advanced Very High Resolution Radiometer
BATC: Ball Aerospace and Technology Corp
BB: Blackbody
CASOTS: Combined Action for the Study of the Ocean Thermal Skin
CEOS: Committee on Earth Observing Satellites
CEOS: WGCV CEOS Working Group on Calibration and Validation
CLARREO: Climate Absolute Radiance and Refractivity Observatory
CME: Coronal Mass Ejections
CrIS: Cross-track Infrared Sounder
CWG: Calibration Working Group – NOAA/NESDIS/STAR/GOES-R
CZCS: Coastal Zone Color Scanner
DMSP: Defense Meteorological Satellite Program
DOD: Department of Defense
DOE: Department of Energy
ECT: External Calibration Target – (alias) Earth Calibration Target
EOS: Earth Observing System
ERBE: Earth Radiation Budget Experiment
ERBS: Earth Radiation Budget Satellite
EUV: extreme ultraviolet
EXIS: EUV and X-Ray Irradiance Sensor
FEL: lamp standard for spectral irradiance
GEOSS: Global Earth Observation System of Systems
GOES: Geostationary Operational Environmental Satellite
GOES-R: Geostationary Operational Environmental Satellite-R Series
GOFR: Gonio Filter Radiometer – NIST
GSFC: Goddard Space Flight Center
GSICS: Global Space-based Inter-Calibration System
HACR: High Accuracy Cryogenic Radiometer
HIRS: High Resolution Infrared Radiation Sounder
ICT: Internal Calibration Target
IPO: Integrated Program Office – NPOESS
ITT: ITT Corporation
JPL: Jet Propulsion Laboratory
JPSS: Joint Polar Satellite System
LBIR: Low Background Infrared (facility)
LUSI: Lunar Spectral Irradiance and Radiance
M-AERI: Marine-Atmosphere Emitted Radiance Interferometer
MBIR: Medium Background Infrared (chamber)
MIT: Massachusetts Institute of Technology
MOBY: Marine Optical Buoy
MOS: Marine Optical System
MOU: Memorandum of Understanding
MSU: Microwave Sounding Unit
NASA: National Aeronautics and Space Administration
NBS: National Bureau of Standards – now NIST
NCC: National Calibration Center at NOAA
NESDIS: National Environmental Satellite, Data and Information Service
NESDIS/ORA: Office of Research and Applications (NESDIS)
NESS: National Environmental Satellite Service
NIST: National Institute of Standards and Technology
NISTIR: NIST Interagency Report
NOAA: National Oceanic and Atmospheric Administration
NPOESS: National Polar-Orbiting Environmental Satellite System
NPP: NPOESS Preparatory Project
NPR: NIST Portable Radiance Source
NRC: National Research Council
OCLI: OCLI, Inc
OTD: Optical Technology Division (NIST) now Sensor Science Division (SSD)
POES: Polar-orbiting Operational Environmental Satellite
PRT: Platinum Resistance Thermometer
PTFE: polytetrafluoroethylene
PTM: ABI Prototype Model
QM-95: Quartz iodine irradiance standard from NBS
RAL: Rutherford Appleton Laboratory
ROLO: Robotic Lunar Observatory – USGS
RSMAS: Rosentiel School of Marine and Atmospheric Science
SBUV: Solar Back Scatter Ultraviolet instrument/2
SeaWiFS: Sea-viewing Wide Field-of-view Sensor
SEM: Space Environment Monitor
SI: International System of Units (Systeme Internationale)
SIRCUS: Spectral Irradiance and Radiance Calibration using Uniform Sources
SLM: Standard Lamp Monitor – NIST
SNO: Simultaneous Nadir Overpasses
S-NPP: Suomi National Polar-orbiting Partnership
SOC: Southampton Oceanography Center
SRF: Spectral Response Function
SSU: Stratospheric Sounding Unit
STAR: Center for Satellite Applications and Research at NOAA/NESDIS
SURF BL-2,7: NIST Synchrotron Ultraviolet Radiation Facility Beam Lines
SUVI: Solar Ultraviolet Imager
SXR: SeaWiFS Transfer Radiometer
TIROS: Television Infrared Observation Satellite
TOVS: TIROS Operational Vertical Sounder
TXR: Thermal-infrared Transfer Radiometer
UCLA: University of California at Los Angeles (UCLA)
USGS: United States Geological Society
VIIRS: Visible/Infrared Imager Radiometer Suite (sensor in S-NPP)
VXR: Visible Transfer Radiometer – NIST
WBBB: Water bath Blackbody
WMO: World Meteorological Organization
